# The role of aquaporin 4 in apoptosis after intracerebral hemorrhage

**DOI:** 10.1186/s12974-014-0184-5

**Published:** 2014-10-31

**Authors:** Heling Chu, Jun Xiang, Pin Wu, Jingjing Su, Hongyan Ding, Yuping Tang, Qiang Dong

**Affiliations:** Department of Neurology, Huashan Hospital, State Key Laboratory of Medical Neurobiology, Fudan University, No. 12 Mid. Wulumuqi Road, Shanghai, 200040 P. R. China; Department of Chinese Integrative Medicine, Zhongshan Hospital, Fudan University, No. 180 Fenglin Road, Shanghai, 200032 P. R. China; Department of Neurology, the Second Hospital of Zhejiang University Medical College, No. 88 Jiefang Road, Hangzhou, 310009 P. R. China; Department of Neurology, Shanghai Ninth People’s Hospital Affiliated Shanghai Jiaotong University School of Medicine, No. 639 Zhizaoju Road, Shanghai, 200011 P. R. China

**Keywords:** apoptosis, aquaporin-4, cytokine, intracerebral hemorrhage

## Abstract

**Background:**

We previously reported that aquaporin-4 deletion (AQP4^−/−^) in mice increased edema and altered blood-brain barrier integrity following intracerebral hemorrhage (ICH). To date, little is known about the role of AQP4 in apoptosis after ICH. The purpose of this study was to examine the role of AQP4 in apoptosis and its mechanisms after ICH using AQP4^−/−^ mice.

**Methods:**

We compared the survival rate and neurological deficits in wild-type (AQP4^+/+^) mice with those in AQP4^−/−^ mice following ICH. Histological changes were detected with terminal deoxynucleotidyl transferase-mediated dUTP-biotin nick end labeling (TUNEL) staining and Hoechst staining. The cell types involved were determined by immunocytochemical studies. We also measured activated caspase-3, caspase-9, caspase-8, Bax, and Bcl-2 with Western blotting at 1, 3, and 7 days after ICH. A cytokine protein assay was used to detect cytokines in AQP4^+/+^ and AQP4^−/−^ mice following ICH, and the results were verified by ELISA.

**Results:**

We found more apoptotic cells in AQP4^−/−^ mice following ICH; the cell types involved were predominantly neurons and astrocytes. Western blotting showed that the expression of activated caspase-3 and caspase-8 was significantly increased (*P* <0.05). Moreover, we demonstrated a greater enhancement in the release of TNF-α and IL-1β, as well as their receptors, in AQP4^−/−^ mice following ICH than in AQP4^+/+^ mice by cytokine protein assay and Western blotting (*P* <0.05). The inhibitors of TNF-α and IL-1β reduced apoptotic cells after ICH in AQP4^−/−^ mice compared with wild-type mice (*P* <0.05).

**Conclusions:**

AQP4 deletion increases apoptosis following ICH, and the underlying mechanism may be through cytokines, especially TNF-α and IL-1β, initiating the apoptotic cascade, as well as activation of caspase-3 and caspase-8.

## Background

Previous evidence indicates that in aquaporin-4 knockout (AQP4^−/−^) mice, the reduced movement of water into and out of the brain results in differing consequences for cytotoxic and vasogenic brain edema [1]. In intracerebral hemorrhage (ICH), however, vasogenic and cytotoxic edema coexist. We previously reported that gene AQP4 deletion in mice increased edema, altered blood-brain barrier integrity, and aggravated neurological deficits following ICH [2]. Moreover, it has also been demonstrated that AQP4 is involved in the protective effects of several neuroprotective factors on ICH [3-5]. These findings provide evidence that AQP4 plays an important role in maintaining water equilibrium, and might even be involved in neuronal protection following ICH.

Apart from its role in edema formation, AQP4 has been reported to be involved in different functions in brain. For example, AQP4 has been identified as a structural component of membrane ‘orthogonal arrays of proteins’, which are cobblestone-appearing structures, seen by freeze-fracture electron microscopy [1]. Furthermore, AQP4 deletion disrupts not only orthogonal arrays of proteins but also gap junctions on the basolateral membranes between adjacent ependymocytes, causing disorganization of the ependyma [6]. In addition, it was also revealed that deletion of AQP4 induced an increase of glutamate levels in the striatum, cortex, and hippocampus, suggesting that AQP4 plays a crucial role in maintaining brain function [7]. Another study also demonstrated that AQP4 deficiency downregulated glutamate uptake and glutamate transporter-1 (GLT-1) expression in astrocytes [8].

Astrocytes are the most abundant type of glial cell in the central nervous system and appear to be involved in the induction of neuroinflammation. Under stress and injury, astrocytes become astrogliotic, leading to upregulation of the proinflammatory cytokines and chemokines, which are associated with apoptosis in a caspase-8-dependent manner [9,10]. To date, however, little is known about the role of AQP4 in astrocyte-derived apoptosis after ICH.

To address this question, we compared survival rate, neurological deficit, terminal deoxynucleotidyl transferase-mediated dUTP-biotin nick end labeling (TUNEL) staining, and Hoechst staining, and determined the cell types involved with immunocytochemical studies between AQP4^−/−^ and wild-type (AQP4^+/+^) mice following ICH. Subsequently, a cytokine protein assay was used to detect cytokines in AQP4^−/−^ and AQP4^+/+^ mice following ICH, and the results were verified by ELISA, to explore the probable mechanisms.

## Methods

### Mice

Male AQP4^+/+^ and AQP4^−/−^ mice, 3 to 4 months old, weighing 25 to 33 g, were kindly provided by Dr. Gang Hu, Jiangsu Key Laboratory of Neurodegeneration, Department of Anatomy, Histology, and Pharmacology of Nanjing Medical University in China. Investigators were blinded to genotype information in all experiments. Mice were kept under environmentally controlled conditions (ambient temperature, 22 ± 2°C; humidity, 40%) on a 12 h light-dark cycle with free access to food and water. All animals were treated according to protocols approved by the Institutional Animal Care and Use Committee of Fudan University.

### Autologous whole blood-induced intracerebral hemorrhage

Mice were anesthetized with 10% chloral hydrate (350 mg/kg) and were placed in a stereotaxic frame (Alcott Biotech, Shanghai, China). Through a hole drilled in the skull, a 32-gage needle was implanted into the striatum, 2.0 mm lateral to the midline, 1.0 mm anterior to the coronal suture, and at a depth of 4.0 mm from the surface of the brain. Each mouse was microinjected with 5 μl of autologous whole blood (right striatum) taken from the tail vein over 10 min using a 5 μl microinfusion pump (ALC-IP600, Alcott Biotech). The needle was then pulled out without blood reflux after 5 min and the wound was sutured. Only mice with neurological deficit were regarded as a successful model and approximately 10% of the total number of mice were excluded, owing to absence of neurological deficit or to unexpected death.

### Experimental groups

This study was divided into nine parts.

1. Two groups (*n* =4 each) of animals (AQP4^+/+^ and AQP4^−/−^ mice) received an intracerebral infusion of 5 μl of autologous whole blood. Physiological values were measured 15 minutes before and 15 and 30 minutes after ICH.
2. Two groups (*n* =24 each) of animals (AQP4^+/+^ and AQP4^−/−^ mice) received an intracerebral infusion of 5 μl of autologous whole blood. Hoechst staining and neurological testing were measured at days 0 (*n* =6), 1 (*n* =6), 3 (*n* =6), and 7 (*n* =6).
3. Two groups (*n* =40 each) of animals (AQP4^+/+^ and AQP4^−/−^ mice) received an intracerebral infusion of 5 μl of autologous whole blood. Mice were observed daily and cumulative mortality was recorded through day 14 post-hemorrhage.
4. Two groups (*n* =48 each) of animals (AQP4^+/+^ and AQP4^−/−^ mice) received an intracerebral infusion of 5 μl of autologous whole blood. Immunocytochemical studies were conducted with antibodies against neuron-specific nuclear protein (NeuN), glial fibrillary acidic protein (GFAP), and TUNEL, to determine the cell types involved. Measurements were made at days 0 (*n* =12), 1 (*n* =12), 3 (*n* =12), and 7 (*n* =12).
5. Two groups (*n* =24 each) of animals (AQP4^+/+^ and AQP4^−/−^ mice) received an intracerebral infusion of 5 μl of autologous whole blood. Western blotting was used to detect apoptosis-related protein expression following ICH. Measurements were made at days 0 (*n* =6), 1 (*n* =6), 3 (*n* =6), and 7 (*n* =6).
6. Two groups (*n* =6 each) of animals (AQP4^+/+^ and AQP4^−/−^ mice) received an intracerebral infusion of 5 μl of autologous whole blood. A cytokine protein assay was used to measure cytokine release in AQP4^−re^ and AQP4^+/+^ mice 3 days (*n* =6) after ICH.
7. Two groups (*n* =24 each) of animals (AQP4^+/+^ and AQP4^−/−^ mice) received an intracerebral infusion of 5 μl of autologous whole blood. ELISA was used to verify measured release of cytokines at days 0 (*n* =6), 1 (*n* =6), 3 (*n* =6), and 7 (*n* =6).
8. Two groups (*n* =48 each) of animals (AQP4^+/+^ and AQP4^−/−^ mice) received an intracerebral infusion of 5 μl of autologous whole blood. After the lateral ventricle was infused with interleukin-1 receptor antagonist (IL-1ra) or tumor necrosis factor binding protein (TNFbp) e, TUNEL staining was measured at days 0 (*n* =12, 6 mice for each inhibitor), 1 (*n* =12), 3 (*n* =12), and 7 (*n* =12).
9. Two groups (*n* =24 each) of animals (AQP4^+/+^ and AQP4^−/−^ mice) received an intracerebral infusion of 5 μl of autologous whole blood. Western blotting was used to measure IL-1β and TNF-α receptor and phosphorylation at days 0 (*n* =6), 1 (*n* =6), 3 (*n* =6), and 7 (*n* =6).

### Survival rate

Mice were observed daily and cumulative mortality was recorded until day 14 post-hemorrhage. Kaplan-Meier survival plots were produced using a log rank test with GraphPad Prism version 3.02 for Windows (GraphPad Software, San Diego, CA, USA). Significance was considered for *P* =0.05 level.

### Measurement of physiological variables

Because the blood volume required for analytic assays was large enough to cause hypovolemia, a separate series was performed to define the physiological state of mice subjected to ICH. In two groups of mice (AQP4^+/+^ and AQP4^−/−^ mice) (*n* =4 each), the right femoral artery was catheterized for continuous blood pressure monitoring and periodic blood sampling for arterial pH, blood gases (PaO_2_, PaCO_2_ ), and glucose (Ciba Corning Diagnostics Corp. East Walpole, MA, USA). Physiological values were measured 15 min before and 15 and 30 min after ICH. These animals thus underwent all acute procedures, including ICH and behavioral testing, and were then killed by an overdose of chloral hydrate anesthesia. All analyses were conducted by an observer blinded to the genotype of the mice.

### Neurological testing

Neurological deficits were evaluated by an observer blinded to the age and genotype of each mouse, including AQP4^+/+^ and AQP4^−/−^ mice, at 1, 3, and 7 days after blood injection. Tests included a postural reflex test, which examined upper-body posture while the animal was suspended by the tail, and a forelimb placing test, which examined sensorimotor integration in the forelimb to visual, tactile, and proprioceptive stimuli. Neurological function was graded on a scale of 0 to 12 (normal score, 0; maximal score, 12), as described previously [11]. Mice that did not demonstrate a right upper extremity paresis during ICH were excluded from further study.

### Brain tissue processing

Both AQP4^+/+^ and AQP4^−/−^ mice were anesthetized with intraperitoneal administration of chloral hydrate. The animals were then perfused transcardially through the ascending aorta, with 20 ml of warm (37°C) 0.9% NaCl containing heparin (10000 U/l) followed by 100 ml of a freshly prepared ice-cold solution of 4% formaldehyde in PBS (0.1 M, pH 7.4). Brains were dissected and separated. The brain tissues were fixed at 4°C in the same fixative for 24 h.

### Immunofluorescence

For immunofluorescence staining, the following antibodies were used: mouse anti-NeuN (1:500; Millipore, Billerica, MA, USA) and rabbit anti-GFAP antibody (1:100; Millipore). Sections were incubated with primary antibodies at 4°C overnight and then incubated with secondary antibodies (1:200) for 90 minutes. Fluorescein isothiocyanate (FITC) coupled goat anti-rabbit IgG, Cy3 coupled goat anti-rabbit IgG, and Cy3 coupled goat anti-mouse IgG (all from Jackson ImmunoResearch, West Grove, PA, USA) were used as appropriate. Primary antibody omission incubations with either blocking solution or PBS were performed, to test the specificity of immunoreactivity. The TUNEL method was used with an *in-situ* cell death detection kit (Roche, Mannheim, Germany) to assess for apoptotic cells. In sections colabeled with TUNEL, a Tdt reaction mix was applied after primary antibodies were rinsed off. The Tdt reaction mix contained biotinylated dNTPs, Tdt buffer, and Tdt enzyme (Gibco, Gaithersburg, MD, USA) in ultrapure H_2_O. After 1 hour (37°C), slides were rinsed in 0.1 M EDTA, pH 8.0 for 2 to 5 minutes, PBS briefly, and then incubated with secondary antibody plus DN-Avidin-FITC (Vector Labs, Burlingame, CA, USA) for 1 h at room temperature. Finally, sections were rinsed in PBS and covered with a cover slip.

For Hoechst staining, sections were stained with Hoechst 33258 (10 minutes), rinsed and cleared in ethanol and xylenes, and covered with a cover slip under Permount (Santa Cruz Biotechnology, Dallas, TX, USA).

### Cytokine protein array analysis

Brain tissues were harvested from AQP4^+/+^ and AQP4^−/−^ mice 3 days after the induction of ICH. Supernatants from brain tissues were analyzed for cytokines using the Mouse Cytokine Array II (RayBiotech, Norcross, GA, USA) according to the manufacturer’s protocol. Briefly, arrayed antibody membranes were incubated with blocking buffer for 30 min at room temperature. Membranes were then probed with 3 ml of conditioned media for 2 h at room temperature. After washing three times with 2 ml of washing buffer, the membranes were incubated with biotin-conjugated anticytokine antibody diluted in blocking buffer for 2 h at room temperature. After the washings, the membranes were developed with an ECL-type system (provided by the kit), exposed to X-ray film (Kodak, Rochester, NY, USA), and processed by autoradiography (Kodak). Autoradiographs of the arrays were scanned to determine the density of the protein array spots and analyzed with a TINA 2.0 program (Raytest, Strasbenhardt, Germany). Relative protein concentrations of different samples were analyzed by comparing densities resulting from subtraction of the blank and normalization for the positive controls.

### Treatment with IL-1ra and TNFbp

The IL-1β inhibitor IL-1ra and the TNF-α inhibitor TNFbp were used. In the appropriate treatment groups, mice were stereotactically injected with inhibitors into the lateral ventricle (0.9 mm lateral to the midline, 0.1 mm posterior to the coronal suture and at a depth of 3.1 mm from the surface of the brain). Injections of 5 μg IL-1ra (Amgen, Thousand Oaks, CA, USA) or 3 mg/kg TNFbp (Amgen Inc., Boulder, CO, USA) were given 30 minutes after induction of ICH. Mice were then killed 1, 3, or 7 days after induction of ICH, and brains were processed for quantification of TUNEL staining.

### Western blotting

Whole brain tissues were homogenized and the samples were loaded onto 4% stacking/12% separating SDS-polyacrylamide gels. The proteins were electrophoretically transferred onto nitrocellulose membranes. After the blocking, the blotting membranes were incubated with specific primary antibodies as follows: rabbit polyclonal against the active cleaved fragment (17 kDa) of caspase-3 (1:1000; Idun Pharmaceuticals, Los Angeles, CA, USA), rabbit polyclonal directed against the active cleaved fragment (20 kDa) of caspase-8 (1:1000, Santa Cruz Biotechnology), rabbit polyclonal directed against the cleaved fragment of caspase-9 (38 kDa) (1:1000, CST, Boston, MA, USA), rabbit polyclonal against Bcl-2, mouse monoclonal against Bax antibodies (1:100, Santa Cruz Biotechnology), rabbit polyclonal against IL-1β receptor and IL-1β phosphorylation receptor (1:1000, CST), and rabbit polyclonal against TNF-α receptor I and TNF-α receptor I phosphorylation (1:1000, CST) for 2 h at room temperature and then with biotinylated goat anti-rabbit IgG (1: 200, Vector Labs) for 1 h at room temperature. The proteins were visualized using the ABC reagent (Vectastain ABC Kit, Vector Labs) and the ECL detection kit (Amersham Pharmacia Biotech, Baie-D’Urfe, QC, Canada). The intensity of blots was quantified using the Leica Image Processing and Analysis System (Frankfurt, Hesse, Germany). β-actin was used as an internal control.

### Cell counting

For cell counting, brain sections of 8 μm thick were cut through the needle entry site, starting with the first appearance of TUNEL-stained or Hoechst-stained cells, extending to the most caudal parts of the hematoma zone, at 1, 3, and 7 days after ICH (*n* =6 per group). Six brain sections per mouse were selected that showed the greatest difference in TUNEL staining or Hoechst staining from the control group. The total hemispheric areas of each section were traced using an image analyzer. Morphometric analysis involved computer-assisted hand delineation of the area of the striatum. Labeled profiles were counted only if the first recognizable profile of the cell soma came into focus within the counting frame. Each of the coronal sections described was used for analysis. Each group included six mice, and six brain sections from each mouse were counted. The mean number of positive cells per section was recorded.

### Statistical analysis

All data were presented as mean ± standard error of the mean. Statistical analysis was performed with two-tailed Student *t* tests or one-way analysis of variance (ANOVA) followed by a post-hoc test for group differences at each time point using SPSS 11.0 for Windows (SPSS Inc, Chicago, IL, USA). Differences were considered significant at *P* <0.05. All analyses were conducted by an observer blinded to the genotype of the mice.

## Results

### Physiological variables between AQP4^+/+^ and AQP4^−/−^ mice

No significant difference was seen in physiological parameters, such as mean arterial blood pressure, PaO_2_, and PaCO_2_ between AQP4^−/−^ mice and AQP4^+/+^ mice before, during, or after ICH. There was also no difference in the levels of serum glucose between AQP4^−/−^ and AQP4^+/+^ mice (Table 1).

**Table 1:** **Physiological variables between AQP4** ^**−/−**^ **and AQP4** ^**+/+**^ **mice**

	**Mean arterial blood pressure (mmHg)**	**pH**	**PaCO**_**2**_ **(mmHg)**	**PaO**_**2**_ **(mmHg)**	**Glucose (mg/dl)**
15 min before intracerebral hemorrhage					
AQP4^−/−^ mice	77.8 ± 10.5	7.35 ± 0.04	43.0 ± 4.5	109.6 ± 11.3	89.9 ± 17.6
AQP4^−/−^ mice	78.6 ± 11.4	7.34 ± 0.03	42.6 ± 2.8	111.4 ± 17.3	91.8 ± 12.7
30 min after intracerebral hemorrhage					
AQP4^−/−^ mice	76.8 ± 12.6	7.36 ± 0.03	43.6 ± 2.6	110.6 ± 173	90.0 ± 16.6
AQP4^−/−^ mice	77.3 ± 12.4	7.35 ± 0.03	43.3 ± 3.0	111.7 ± 16.5	91.3 ± 15.4

PaO_2_, partial pressure of arterial oxygen; PaCO_2_, partial pressure of arterial carbon dioxide. Blood gas parameters (pH, PaCO_2_ and PaO_2_) and glucose level were recorded in blood samples of mice (*n* =4). Data are means ± standard deviation. None of the differences were achieved, or showed a trend towards, statistical significance.

### Survival rate

**Figure 1:**
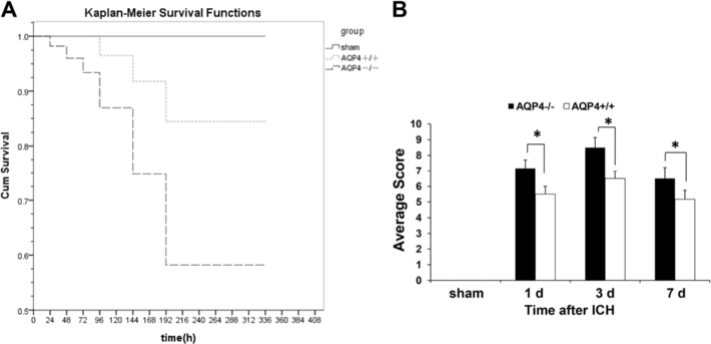
**Survivor function of each group of AQP4** ^**−/−**^ **and AQP4** ^**+/+**^ **mice. (A)** Kaplan − Meier plots illustrate survival curves, showing a higher survival rate in AQP4^+/+^ mice than in AQP4^−/−^ groups by day 14 after ICH. **(B)** ICH-induced neurological deficits were observed. It was demonstrated that AQP4 deletion significantly worsened neurological impairments at 1, 3, and 7 days after ICH (*n* =6, * *P* <0.05). ICH, intracerebral hemorrhage.

The KaplanMeier plots in Figure 1A illustrate the survival curves for AQP4^−/−^ and wild-type mice. The survival curve for AQP4^−/−^ mice group showed a mortality of 42.5% by day 14 after ICH, with only 23 mice surviving to the end of the study. In contrast, wild-type mice had a higher survival rate than AQP4^−/−^ mice, with 85% (34 mice) surviving to the end of the study (*P* <0.05). In addition, initial deaths for wild-type mice groups did not occur until 96 h after ICH, while for AQP4^−/−^ mice, initial deaths were advanced to 24 h after ICH. These results indicated that AQP4 significantly increased survival rate of mice undergoing ICH.

### Higher neurological scores of AQP4^−/−^ mice than of AQP4^+/+^ mice following intracerebral hemorrhage

The AQP4 deletion worsened the neurological impairment after ICH. Both AQP4^−/−^ mice and AQP4^+/+^ mice began to exhibit neurological signs 1 day after ICH, which lasted for at least 7 days (*P* <0.05). Neurological scores of AQP4^−/−^ mice were higher than those of AQP4^+/+^ mice at all time points (*P* <0.05) (Figure 1B).

### More apoptosis in AQP4^−/−^ mice than in AQP4^+/+^ mice following intracerebral hemorrhage

**Figure 2:**
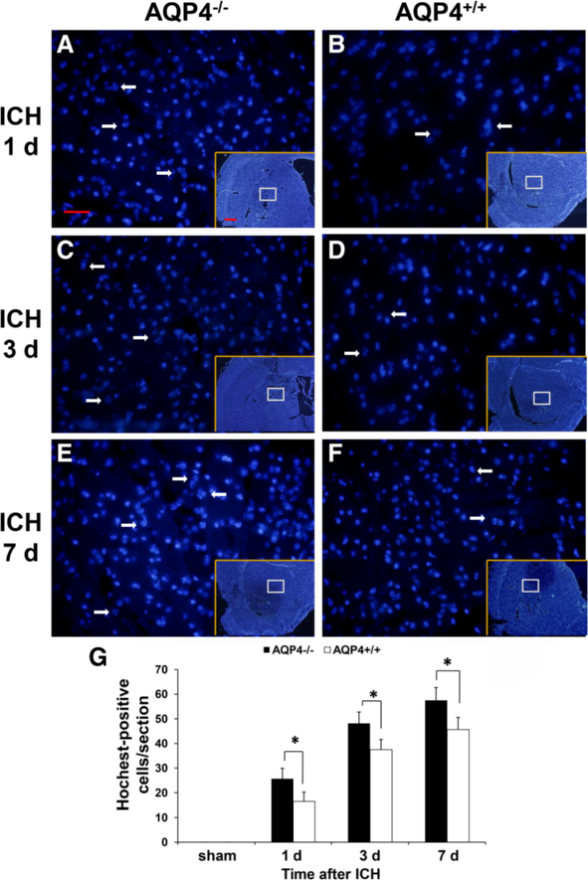
**Hoechst staining of AQP4** ^**−/−**^ **and AQP4** ^**+/+**^ **mice. (A-F)** Hoechst-positive cells with morphologies suggestive of apoptosis showed dense granule fluorescence in the nucleus from AQP4^−/−^ and AQP4^+/+^ mice, which represent DNA fragment (arrows). Scale bar: 50 μm (larger graph); 500 μm (lower right graph) **(G)** Quantitative analysis of Hoechst-positive cells at 1, 3, and 7 days after ICH (*n* =6, * *P* <0.05). ICH, intracerebral hemorrhage.

**Figure 3:**
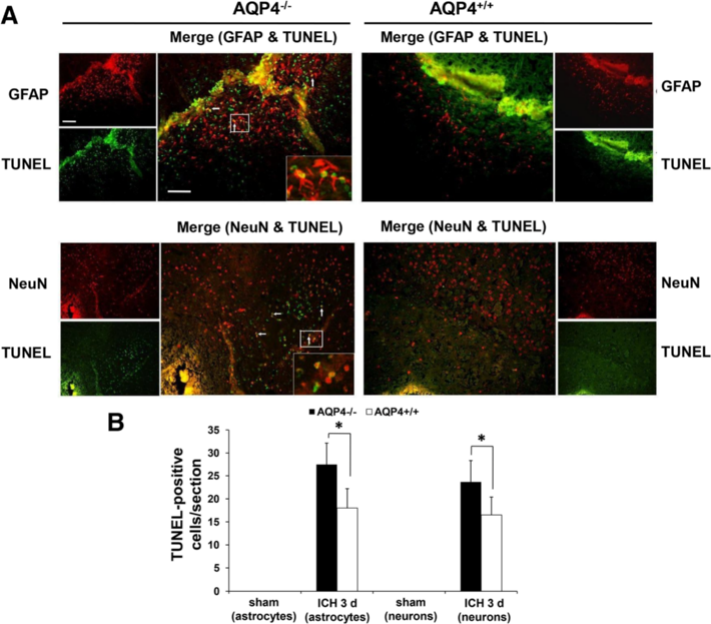
**Representative TUNEL-positive cells with morphologies suggestive of apoptosis. (A)** Left, AQP4^−/−^ mice; right, AQP4^+/+^ mice. Colocalized cells (yellow, arrows) by TUNEL (green) and NeuN (red) fluorescence represent the cell type as neuron, while those double labeled by TUNEL (green) and GFAP (red) fluorescence are astrocytes. Magnified images of the regions of interest are indicated by a white rectangle in the frame at the lower right corner. Scale bar: 500 μm (left graph); 200 μm (right graph). **(B)** Quantitative analysis shows more apoptosis-positive cells (both neurons and astrocytes) 3 days after ICH in AQP4^+/+^ mice than in AQP4^−/−^ mice (*n* =6, * *P* <0.05). GFAP, glial fibrillary acidic protein; ICH, intracerebral hemorrhage; NeuN, neuron-specific nuclear protein; TUNEL, terminal deoxynucleotidyl transferase-mediated dUTP nick end labeling.

To explore the cause of neurological deficit aggravation in AQP4^−/−^ mice, we also detected apoptosis via Hoechst staining (Figure 2) and TUNEL staining (Figure 3). Our experiments showed that AQP4 deletion promoted Hoechst staining and TUNEL staining after ICH. The Hoechst staining and TUNEL staining were markedly increased at 1, 3, and 7 days after ICH in both groups. More Hoechst staining and TUNEL staining in AQP4^−/−^ mice was observed than in AQP4^+/+^ mice. The TUNEL-positive cells were principally seen at hematoma zone from AQP4^+/+^ mice after ICH (Figure 3A). Immunocytochemical studies were conducted with antibodies against neurons (NeuN) and astrocytes (GFAP) to determine the cell types involved. Fluorescent microscopy showed that the majority of the TUNEL-positive cells were colocalized with these markers for neurons and astrocytes (Figure 3A). In Figure 3B, quantitative results of merged cells showed that AQP4 deletion promoted TUNEL staining after ICH (*P* <0.05).

### Higher levels of apoptosis-related proteins in AQP4^−/−^ mice than in AQP4^+/+^ mice following intracerebral hemorrhage

**Figure 4:**
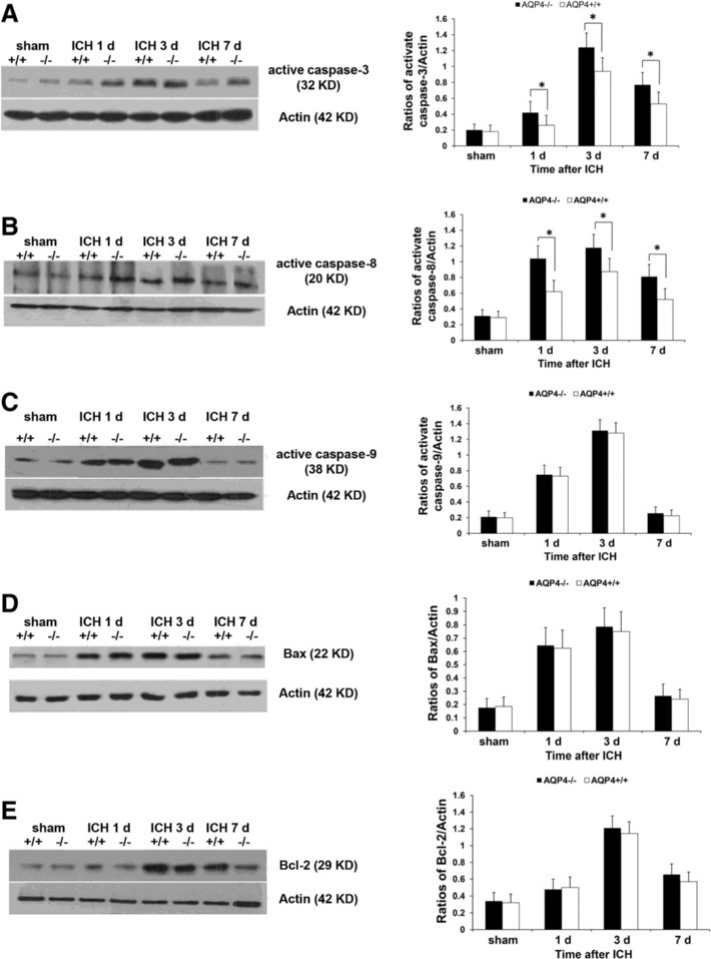
**Expression of apoptosis-related proteins in AQP4** ^**−/−**^ **and AQP4** ^**+/+**^ **mice.** Higher levels of cleavage (activated) caspase-3 and caspase-8 were demonstrated at 1, 3, and 7 days after ICH in AQP4^−/−^ mice than in AQP4^+/+^ mice by Western blotting (*n* =6, * *P* <0.05). However, no changes of active caspase-9, Bax, or Bcl-2 were observed at any time point. **(A)** Expression of active caspase-3. **(B)** Expression of active caspase-8. **(C)** Expression of active caspase-9. **(D)** Expression of Bax. **(E)** Expression of Bcl-2. ICH, intracerebral hemorrhage.

To explore the mechanisms of neuronal apoptosis induced by AQP4 deletion, we measured expression of apoptosis-related proteins. Western blotting revealed that the levels of activated caspase-3 and caspase-8 were significantly increased from 1 day to 7 days after ICH in both AQP4^−/−^ and AQP4^+/+^ mice (*P* <0.05). Higher levels of caspase-3 and caspase-8 were detected in AQP4^−/−^ mice than in wild-type mice following ICH (*P* <0.05). However, Western blotting showed that caspase-9, Bax, and Bcl-2 had no significant difference between the two types of mice after ICH (Figure 4).

### Enhanced release of cytokines in AQP4^−/−^ mice than that in AQP4^+/+^ mice following intracerebral hemorrhage

**Figure 5:**
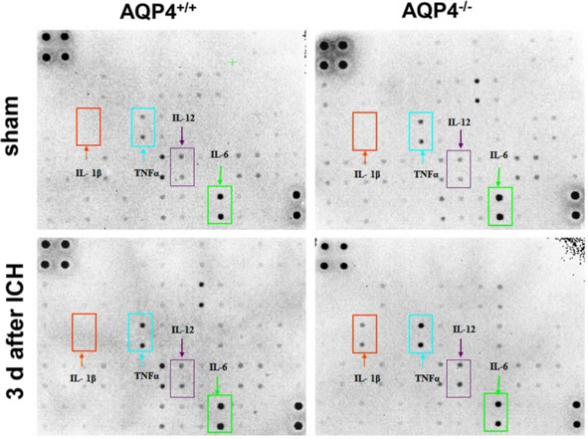
**Release of cytokines in AQP4** ^**−/−**^ **and AQP4** ^**+/+**^ **mice.** Mouse cytokine array was used in this section. The odds differences from scans of AQP4^−/−^ and wild-type mice with or without ICH were adjusted for the intensity of positive controls on the filter corners and plotted for comparison between the two groups. A greater enhancement of release of IL-1β, TNF-α, IL-6 and IL-12 was detected in AQP4^−/−^ mice than AQP4^+/+^ mice 3 days after ICH. ICH, intracerebral hemorrhage.

**Figure 6:**
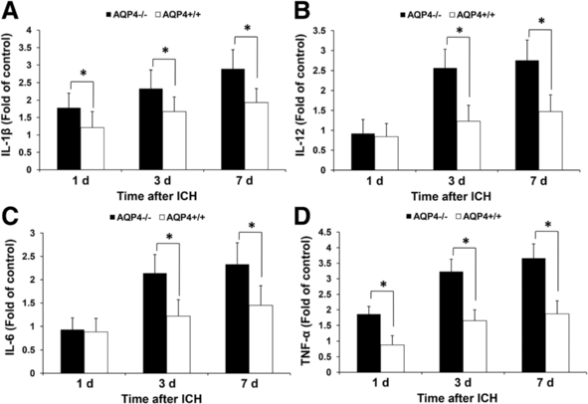
**Quantitative analysis of the expression of cytokines.** Expression of TNF-α and IL-1β was significantly increased from 1 day to 7 days in both AQP4^−/−^ and AQP4^+/+^ mice after ICH, while upregulation of IL-6 and IL-12 was only detected at 3 days and 7 days after ICH (*n* =6, * *P* <0.05). **(A)** Expression of IL-1β. **(B)** Expression of IL-12. **(C)** Expression of IL-6. **(D)** Expression of TNF-α. ICH, intracerebral hemorrhage.

As summarized in Table 2, a greater enhancement in the release of 4 (IL-6, IL-12, TNF-α, and IL-1β) of the 12 cytokines was demonstrated in AQP4^−/−^ mice at 3 days after ICH. These results were detected by cytokine protein array (Figure 5) and verified by ELISA (Figure 6). Expression of TNF-α and IL-1β was significantly increased from 1 day to 7 days in both AQP4^−/−^ mice and AQP4^+/+^ mice after ICH, while upregulation of IL-6 and IL-12 was only detected at 3 days and 7 days after ICH (*P* <0.05). Higher levels of cytokines were seen in AQP4^−/−^ mice than in wild-type mice following ICH (*P* <0.05).

**Table 2:** **Enhanced release of cytokines in AQP4** ^**−/−**^ **mice than in AQP4** ^**+/+**^ **mice following intracerebral hemorrhage**

	**Mouse cytokine antibody array**	**Fold increase**
IL-1β	Interleukin-1 β	2.1
TNFα	Tumor necrosis factor α	2.3
IL-6	Interleukin-6	1.65
IL-12	Interleukin-12	1.62
bFGF	Basic fibroblast growth factor	−2.2
VEGFRI	Vascular endothelial growth factor receptor 1	−1.8
DPPIV/CD26	Dipeptidyl peptidase IV	−1.72
Dtk	Dtk	−1.76
E-seletin	Endothelial leukocyte adhesion molecule-1	−1.65
FcγR II B	Human immune globulin Fc receptor	−1.82
GITR	Glucocorticoid induced tumor necrosis factor receptor	−1.68
TSLP	Human thymic stromal lymphopoietin	−1.74

Quantitative analysis of the release of 12 cytokines 3 days after intracerebral hemorrhage. Values are means of two independent experiments, statistically different between AQP4^−/−^ and AQP4^+/+^ mice.

### TUNEL staining in AQP4^−/−^ mice was reduced by IL-1ra or TNFbp

**Figure 7:**
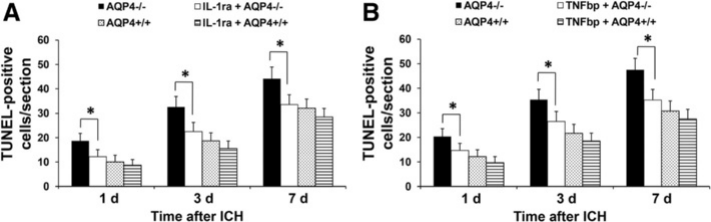
**Effect of IL-1ra and TNFbp on apoptosis in AQP4** ^**−/−**^ **and AQP4** ^**+/+**^ **mice.** Intracerebroventricular injection of IL-1ra and TNFbp significantly reduced TUNEL staining in AQP4^−/−^ mice at 1 d, 3 d and 7 d after ICH (n =6, * *P* <0.05), while they had no effect on apoptosis in AQP4^+/+^ mice. **(A)** Effect of IL-1ra. **(B)** Effect of TNFbp. ICH, intracerebral hemorrhage.

We further used inhibitors to determine the effect of IL-1β and TNF-α on apoptosis following ICH. The untreated mice subjected to ICH were compared with mice treated with IL-1ra or TNFbp. In AQP4^−/−^ mice treated with the inhibitors, the TUNEL staining was significantly reduced at 1, 3, and 7 days after ICH (*P* <0.05), while the inhibitors had no effect on apoptosis in AQP4^+/+^ mice (Figure 7).

### Higher levels of IL-1β and TNF-α receptors in AQP4^−/−^ mice than in AQP4^+/+^ mice following intracerebral hemorrhage

**Figure 8:**
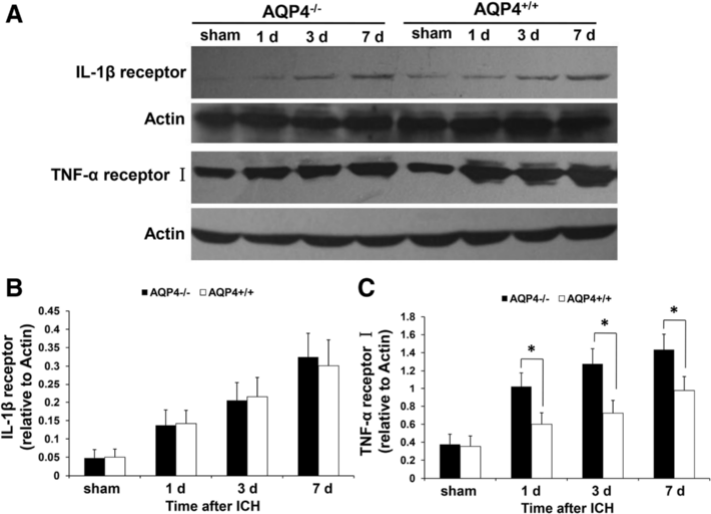
**Expression of IL-1 β and TNF-α receptors in AQP4** ^**−/−**^ **and AQP4** ^**+/+**^ **mice.** It was revealed by Western blotting that higher levels of IL-1β and TNF-α receptors were detected in AQP4^−/−^ mice than in AQP4^+/+^ mice at 1, 3, and 7 days after ICH (*n* =6, * *P* <0.05). **(A)** Western blotting images. **(B)** Semiquantitative analysis of IL-1β receptor. **(C)** Semiquantitative analysis of TNF-α receptor I. ICH, intracerebral hemorrhage.

Western blotting revealed that higher levels of IL-1β and TNF-α receptors were detected in AQP4^−/−^ mice than in AQP4^+/+^ mice at 1, 3, and 7 days after ICH, indicating that AQP4 deletion not only increased IL-1β and TNF-α, but also induced upregulation of their receptors (*P* <0.05) (Figure 8).

### Higher levels of IL-1β and TNF-α receptors phosphorylation in AQP4^−/−^ mice than in AQP4^+/+^ mice following intracerebral hemorrhage

**Figure 9:**
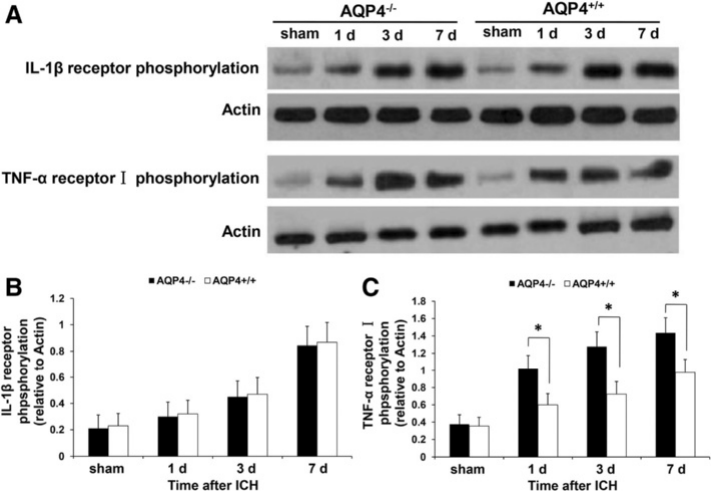
**Expression of IL-1 β and TNF-α receptors phosphorylation in AQP4** ^**−/−**^ **and AQP4** ^**+/+**^ **mice.** It was demonstrated that there were higher levels of IL-1β and TNF-α receptors phosphorylation in AQP4^−/−^ mice than AQP4^+/+^ mice at 1, 3, and 7 days after ICH by Western blotting (*n* =6, * *P* <0.05). **(A)** Western blotting images. **(B)** Semiquantitative analysis of IL-1β receptor phosphorylation. **(C)** Semiquantitative analysis of TNF-α receptor I phosphorylation. ICH, intracerebral hemorrhage.

Similarly, besides the receptors, it was also demonstrated that there were higher levels of IL-1β and TNF-α receptors phosphorylation at all time points after ICH by Western blotting (*P* <0.05) (Figure 9).

## Discussion

It is known that cell death after ICH is mediated in part by apoptotic mechanisms [12,13]. In this study, AQP4^−/−^ mice showed worsened neurological impairment after ICH. To explore the mechanisms of neurological deficit aggravation in AQP4^−/−^ mice, we detected cell apoptosis via Hoechst staining and TUNEL staining, and the results showed that AQP4 deletion promoted cell apoptosis after ICH. Meanwhile, we identified, at 1, 3, and 7 days after ICH, that large numbers of TUNEL-positive stained cells with morphologies suggestive of apoptosis surrounding the hematoma. Double staining with TUNEL and immunocytochemical labeling with antibodies against NeuN or GFAP suggested that these apoptotic cells were mostly neurons and astrocytes. Results from this study provided evidence that apoptosis might play an important role in the pathophysiology of cell death in AQP4^−/−^ mice after ICH.

As known, two pathways of apoptosis have been identified, the ‘extrinsic’ and ‘intrinsic’ pathways [14]. Induction of the extrinsic pathway of apoptosis is associated with the activation of extracellular TNF superfamily cell death receptors; these receptors then recruit other proteins to form a complex that activates caspase-8, which in turn activates caspase-3 [15]. By contrast, in the intrinsic apoptosis pathway, the mitochondrion plays a crucial role by releasing cytochrome c into the cytosol from the mitochondrial intermembrane space. Cytochrome c release, which is initiated by Bax translocation to the mitochondrial membrane and modulated by competition with Bcl-2, an apoptosis inhibitor [16,17], results in forming the apoptosome complex that activates caspase-9, and this in turn activates the executioner caspase-3 [18]. We partially investigated both extrinsic and intrinsic pathways in our work and found that the levels of activated caspase-3 and caspase-8 were markedly lower in AQP4^+/+^ mice than in AQP4^−/−^ mice at 1, 3, and 7 days after ICH by Western blotting. However, other apoptosis-related proteins, caspase-9, Bax and Bcl-2, had no significant difference between AQP4^−/−^ and AQP4^+/+^ mice after ICH, indicating that the extrinsic pathways of apoptosis seem to be mainly involved in neuronal apoptosis induced by AQP4 deletion. However, the triggers that are responsible for initiating the apoptotic cascade following ICH remain to be defined.

AQP4 is the main water channel in the brain and is strongly expressed in astrocyte plasma membranes. Studies have indicated the key roles of AQP4 in astrocyte swelling, growth and migration, and these roles may explain the reason for more astrocyte apoptosis in AQP4^−/−^ mice [19,20]. However, the relationship between AQP4^−/−^ and increased neuronal apoptosis remains to be defined. Astrocytes provide structural, trophic, and metabolic support to neurons and modulate synaptic activity. During brain insult, the reaction of astrocytes is similar in some respects to the inflammatory response of peripheral tissues [21]. This response may be adaptive in settings such as infection, but may contribute to delayed neuronal death in settings such as stroke.

Astrocytes stimulated by injuries produce many cytokines, including TNF, IL, and interferons. It is known that a wide variety of cytokines activated after vascular injury can lead directly to signaling pathways that mediate apoptosis [22]. Although there are probably a multitude of potential cytokines or other factors that might be activated or released following ICH, several of these may be considered to be leading candidates for this role. In this study, cytokine assay indicated that AQP4 deletion induced an enhancement in the release of IL-6, IL-12, TNF-α, and IL-1β, especially the latter two, following ICH. AQP4 has a close relation with proinflammatory cytokines. These cytokines can induce AQP4 gene and protein expression [23]. However, little is known about the effects of AQP4 deletion on cytokine release. It was demonstrated that AQP4 knockout increased protein kinase C (PKC) activity and that PKC inhibitors reduced proinflammatory activity in ischemic tissue, indicating that AQP4 deletion might result in upregulation of proinflammatory cytokines via the PKC pathway [24,25].

In addition, Western blotting indicated that AQP4 deletion induced higher levels of TNF-α and IL-1β receptor expression and phosphorylation, in accord with the proteins. Further treatment with IL-1ra and TNFbp significantly reduced the level of TUNEL staining in AQP4^−/−^ mice, indicating that TNF-α and IL-1β might be two causes of cell apoptosis after ICH in AQP4^−/−^ mice. Apart from TNF-α, mentioned previously, IL-1β is also reported to promote apoptosis and contribute to caspase-3 activation [26,27]. Therefore, we speculate that the key factors initiating apoptotic cascade induced by AQP4 deletion might be the two cytokines.

## Conclusions

Our results suggest that AQP4 deletion worsens brain damage via increasing apoptosis (mainly neurons and astrocytes) following ICH. The mechanism may be ascribed to excessive expression of cytokines, especially IL-1β and TNF-α, initiating apoptotic cascade; activation of apoptosis-related proteins caspase-3 and caspase-8 is also involved. However, our research has some limitations. We demonstrated that AQP4 deletion has a close relation with apoptosis after ICH, but we have not comprehensively investigated the underlying mechanisms, especially the definite entire pathway, and whether it is extrinsic or intrinsic. Meanwhile, the signal pathways through which AQP4 deletion leads to proinflammatory factors release and apoptosis initiation have not been studied. Therefore, our further investigation should focus on these limitations. We also need to explore the effects of AQP4 deletion on apoptosis using cell culture models to obtain more evidences. These findings may provide therapeutic target for ICH-induced brain injury.

## Abbreviations

ANOVA: analysis of variance
AQP4: aquaporin-4
ELISA: enzyme-linked immunosorbent assay
FITC: fluorescein isothiocyanate
GFAP: glial fibrillary acidic protein
GLT-1: glutamate transporter-1
ICH: intracerebral hemorrhage
IgG: immunoglobulin G
IL: interleukin
IL-1ra: interleukin-1 receptor antagonist
NeuN: neuron-specific nuclear protein
PBS: phosphate-buffered saline
PKC: protein kinase C
TNF: tumor necrosis factor
TNFbp: tumor necrosis factor binding protein
TUNEL: terminal deoxynucleotidyl transferase-mediated dUTP nick end labeling
